# Supplementary material for: Cell-connectivity-guided trajectory inference from single-cell data
Source: Bioinformatics. 2023 Aug 25;39(9):btad515. doi: 10.1093/bioinformatics/btad515 (PMC10474950; doi:10.1093/bioinformatics/btad515)
Supplement: btad515_Supplementary_Data [file btad515_supplementary_data.pdf]

Supplementary Information

Supplementary Figure 1

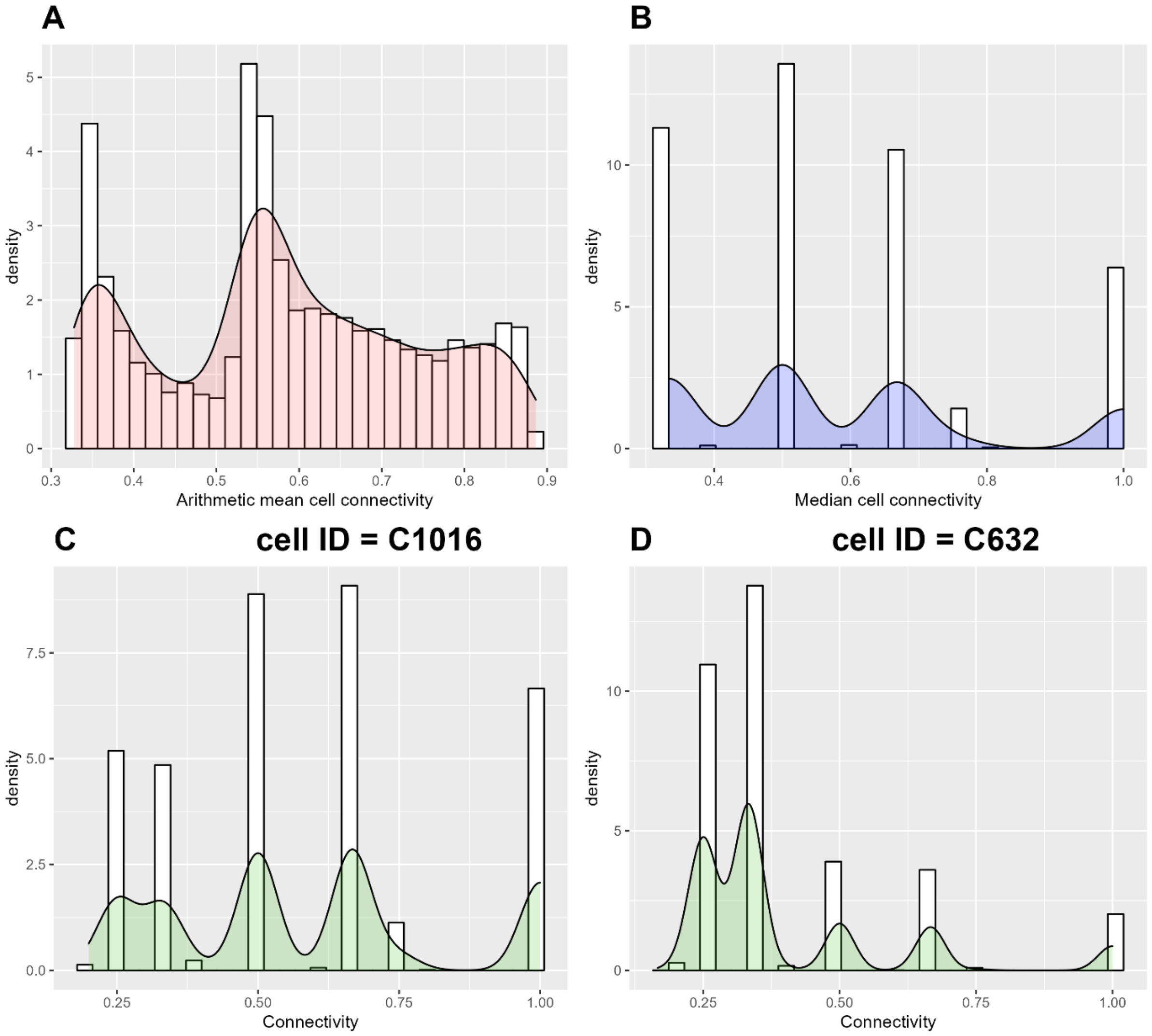

***Supplementary Figure 1**: **Comparing the mean and median approaches for calculating cell connectivity**. A cell connectivity value was calculated for each cell from the multifurcating_4 dataset, which is the dataset used in **Fig. 4A**. For the 10,000 connectivity vectors generated based on 10,000 Minimum Spanning Trees (MST), we aggregated the connectivity values using **(A)** the arithmetic mean and **(B)** the median. **(C-D)** Visualization of the cell connectivity values for two randomly chosen cells.*

Supplementary Figure 2

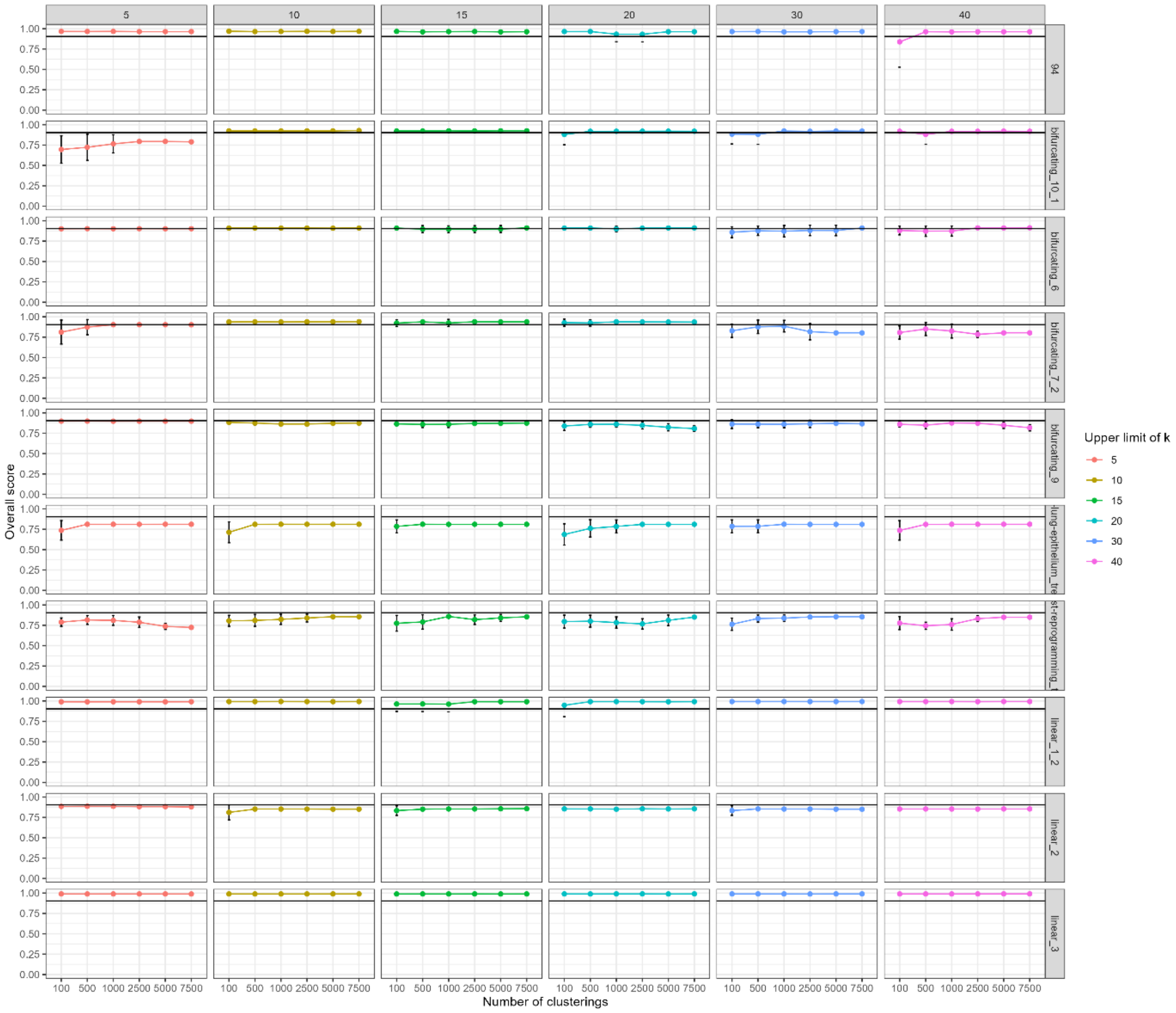

***Supplementary Figure 2**: **Dataset-level robustness of Totem performance**. To investigate Totem's robustness, we varied the number of clusterings (x-axes) and the upper limit of the number of clusters (k) per clustering (column panels) and investigated the overall score in ten datasets (row panels). The ten datasets were randomly selected from Totem's top 50 highest-performing datasets in the dynverse evaluation. The solid horizontal line depicts the average performance of the ten datasets obtained using the default settings (10,000 clusterings, upper limit of 20). For each of the ten datasets, we repeated the Totem analysis with ten different random seeds. The error bars depict the standard deviation across the repeats. The dataset names in the row panels are the original names from the dynverse benchmark data.*

Supplementary Figure 3

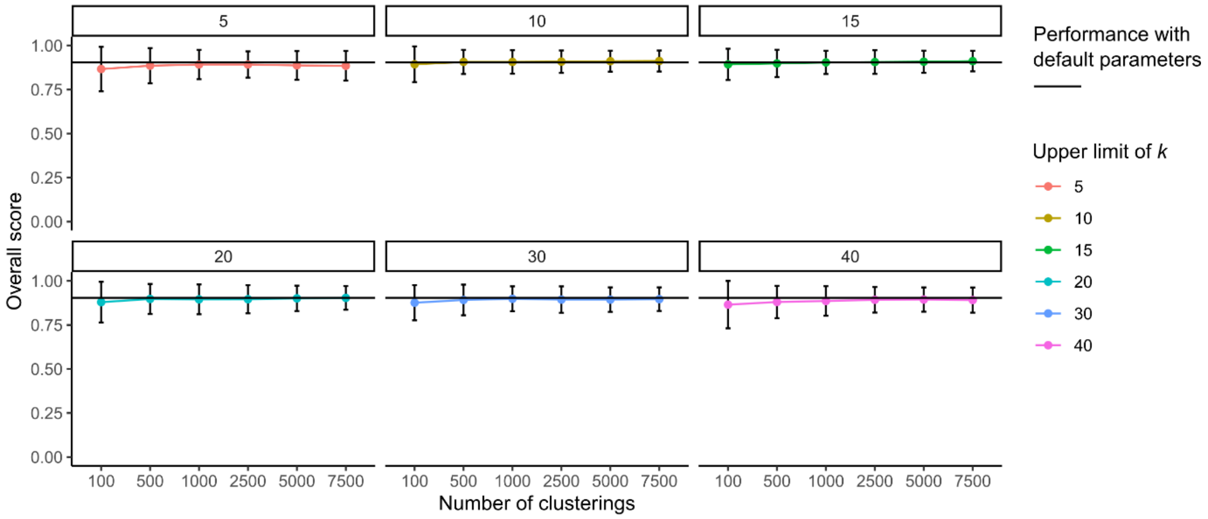

***Supplementary Figure 3**: **Average robustness of Totem performance**. To investigate Totem's robustness, we varied the number of clusterings and the upper limit of the number of clusters (k) and investigated their impact on the overall score in ten datasets. The ten datasets were randomly selected from Totem's top 50 highest-performing datasets in the dynverse benchmark. The solid, horizontal line depicts the average performance of the ten datasets obtained using the default settings (10,000 clusterings, upper limit of 20). For each of the ten datasets, we performed Totem analysis with ten different random seeds, generating ten sets of dissimilar clusterings and trajectories for each dataset. Each parameter configuration hence comprises 100 overall score values from the different datasets and random seeds. The overall score is the geometric mean of the four main performance metrics in the dynverse benchmark. The error bars depict the standard deviation. **Supplementary Figure 2** visualizes the performance at the dataset level.*
